# Pain Management in Oncology Patients Amidst the Opioid Epidemic: How To Minimize Non-Medical Opioid Use

**DOI:** 10.7759/cureus.19500

**Published:** 2021-11-12

**Authors:** Michael Chahin, Sabrina Matosz, Irene Khalel, Silas Day, Amany Keruakous

**Affiliations:** 1 Department of Hematology and Oncology, Georgia Cancer Center at Augusta University, Augusta, USA; 2 Internal Medicine, University of Alexandria, Faculty of Medicine, Alexandria, EGY; 3 Hematology and Medical Oncology, University of Oklahoma Health Sciences Center, Oklahoma City, USA

**Keywords:** oncology, non-medical opioid use, opioid epidemic, pain related to neoplasm, multi-modal treatment approach

## Abstract

The opioid epidemic continues to be a significant public health concern. On the surface, it appears difficult to understate the profound consequences and unnecessary loss of life at the hands of the opioid crisis throughout the 2010s. This reality should not dissuade rigorous attention toward those who have suffered unnecessarily due to an overreaching backlash toward the opioid crisis.

Oncology patients have been significantly impacted on both ends of the opioid crisis. Like other populations, cancer patients were first affected during the initial surge of opioid availability, prescription, and use at the beginning of the crisis, where opioid abuse and overdose negatively impacted cancer patient populations at similar rates as the general population. Yet, cancer patients were perhaps even more heavily affected during secondary events after the initial crisis, as opioid restrictions and the stigmatization, undoubtedly beneficial in many spaces, of opioid use became prevalent across the American society. During this second period of the opioid crisis (loosely from 2013 to the present day after the Veterans Health Administration Opioid Safety Initiative started), restrictions on opioids have significantly decreased the use and access of opioids for cancer patients.

Management of pain, in general, is a complex topic, and cancer pain is no exception. Cancer patients may experience pain related to the disease itself, its treatment, or other comorbidities. This review aims to clarify the impact of reducing opioid use on cancer patients over the past eight years. We summarize the challenges facing providers as they attempt to manage cancer-related pain. Additionally, we propose tools for best practices to reduce the unnecessary suffering of cancer patients and protect against the overuse and abuse of opioids.

## Introduction and background

The opioid crisis continues to be a significant public health concern. According to the Centers for Disease Control and Prevention (CDC), in 2019, there were nearly 50,000 deaths attributed to opioid overdose in the United States [1]. Although opioid-related deaths are more prominent in the general population compared to the cancer patient population [2], there is an increased prevalence of opioid use among cancer patients, and the rate of opioid abuse is approximately similar between the two populations [3-6].

In the era of novel targeted therapeutics and immunotherapy with the increasingly recognized diseases and diagnosing patients in the earlier stages of incurable cancers, the need for adequate pain management is expected as many patients experience a more chronic course. Currently, in the United States, there are approximately 15.5 million cancer survivors, and, of these, more than two-thirds have lived five years or more after their diagnosis, and 44% have survived 10 years or more [7].

Globally, 8.2 million people die of advanced cancer each year. Interestingly, World Health Organization (WHO) estimates that around 6 million of these patients have inadequate or no access to strong opioids because of no increase in the availability of opioids for decades among the world’s poorest but most populated countries due to strict government regulations [8,9].

Even in developed countries, at least 32% of cancer patients remain undertreated due to concern about abuse and increased restrictions of prescribing regulations for opioids [10,11]. Only 43-48% of UK cancer patients receive a potent opioid before their death. A study demonstrated that the median time between initiation of opioids and death for 6,080 patients was nine weeks [12].

This restrictive attitude toward opioids exists due to the potential harms of these drugs; however, an extensive systematic review of 52 studies confirmed that 53% of people at all stages of cancer experienced pain, affecting approximately 64% of patients with advanced cancer [13]. Oncology patients have access to opioid therapy relatively late in their disease course and are more likely to receive opioids in the hospital setting. Postponing the initiation of opioid therapy for cancer-related pain could cause more hospital admissions and worse patient outcomes [14]. Initiating opioid treatment for cancer-related pain is an effective intervention resulting in reasonable pain control in 75% of patients.

Despite the known efficacy of opioids in relieving cancer-related pain, routine screening for the risk of aberrant opioid behaviors is not performed in common clinical practice. Limited published data on the frequency of aberrant drug use make this challenging for prescribers. This obstacle limits clinicians’ ability to determine the frequency and factors predicting the risk of drug misuse behaviors among patients in the outpatient setting, limiting pharmacologic therapies prescribed. Moreover, some formulations may aggravate the potential harms of addiction and mortality with other medications patients may be on such as benzodiazepines [15].

Management of pain, in general, is complex and cancer pain is no exception. Cancer patients may experience pain related to the disease itself, treatment, or from other comorbidities [16]. Opioid therapy is the backbone in managing moderate-to-severe cancer-related pain [13,17]. Therefore, clinicians caring for cancer patients must recognize that the challenges associated with opioid use and misuse, seen in the general population, are also present in this cohort.

Identifying the impact of opioid use on cancer patients is crucial; it is prudent to review the progress made in tackling the opioid crisis. In the United States, the 2000s and early 2010s marked a sharp increase in adverse outcomes related to opioids. This culminated in medication-focused guidelines such as the Veterans Health Administration Opioid Safety Initiative (VHA, OSI) and the CDC Guideline for Prescribing Opioids for Chronic Pain. Since then, there has been a decrease in the overall number of opioid prescriptions. Likely, this can be attributed to not only educating prescribers on this issue but also raising public awareness [18]. There is evidence that this reduction in opioid prescriptions has been demonstrated in both oncologists and non-oncologists between 2013 and 2017 [19].

On the surface, reducing opioid prescriptions is encouraging, but it begs the question of whether cancer pain is adequately treated. It is important to note that the opioid prescribing guidelines by the CDC, for example, specifically do not pertain to cancer patients [20]. A retrospective cohort study of 42,064 opioid naïve Veteran Affairs patients from 2011 to 2016 showed a decrease in new, persistent, and specific high-risk opioid prescribing following the launch of the VHA OSI in 2013. There was also an increase in pain-related emergency department visits [21,22].

Therefore, the purpose of this review article is to identify the challenges facing the oncology population regarding opioid therapy. Additionally, we aim to provide a framework for treating cancer-related pain by reviewing tools useful for assessing cancer pain and general principles for prescribing, titration, maintenance, and safety of opioid use among cancer patients.

## Review

Definitions and terminology of cancer-related pain

It is essential to be familiar with the terminology of opioid abuse or, more appropriately, opioid use disorder. The CDC defines *opioid use disorder* as a pattern of opioid use leading to significant impairment or distress. *Non-medical use* includes using these prescription drugs by someone other than the intended patient and/or deviating from the deliberate regimen or reason. To achieve an intentional response, one must also identify if a patient has opioid tolerance, increasing doses to achieve the required effect [20]. Opioid dependence has multiple criteria, including the compulsion to use opioids, difficulty controlling opioid use, and experiencing withdrawal [23,24].

Screening for opioid misuse

Screening can help clinicians tailor treatment options and monitoring strategies for patients. It is also essential to identify patients at risk of substance dependence. Genetics, drug availability, peer substance use, social norms about substance use, family history of drug use, and poor educational attainment are risk factors associated with opioid dependence.

Opioid therapy is an effective treatment for pain among many patients, especially oncology patients. However, opioid misuse puts the patients at risk for addiction, overdose, and non-medical opioid use (NMOU). Using high opioid doses is more acceptable among oncology patients with short life expectancy than patients with non-malignant pain [25,26].

Risk factors of NMOU include a family history of drug abuse, poor social support, personal history of drug abuse, history of sexual abuse, and psychiatric illnesses such as major depression, bipolar disorder, or personality disorders [25,27-29].

Patients with chronic pain and a history of substance use may develop substance use disorder. Some patients with no prior substance use history may develop aberrant drug-related behaviors [29]. Assessment tools can be utilized to help follow the patients and prevent NMOU. Biological assessment includes history; examination; pain intensity and location; social assessment such as home, work, and social functioning; and psychological assessment such as coping skills, addiction history, or personality disorders [30].

Proposed assessment tools

Cancer pain comprises multiple pain syndromes with different etiologies. This pain may be a consequence of the malignancy itself, therapeutic interventions, or other comorbidities. No pain classification system is universally accepted as a standard. An example is the Edmonton Classification System for Cancer Pain. This tool can assess the complex nature of cancer pain by prompting the practitioner to review, albeit simplistically, the mechanism of the pain, whether addictive behavior is present, and the impact the pain is having psychologically and cognitively. In general, when assessing cancer pain, one should incorporate pain intensity, which can be measured on a scale of 1-10, the location, timing, and temporal variation. Timing distinguishes the pain as acute or chronic, with the caveat that it can be challenging to distinguish the two. Temporality assesses whether the pain is intermittent or continuous. The latter category serves to identify breakthrough pain episodes that can occur over a background pain level [16,31].

Though all assessment tools are imperfect, the details of the proposed assessment tools remain consequential. Uncontrolled cancer pain can lead to psychosocial consequences and poor quality of life. It can also cause distress and hopelessness and can impact a patient’s compliance with a treatment plan [32]. When assessing cancer pain, one must review the reported symptoms and their impact on a patient’s quality of life.

Psychiatric diagnoses, substance misuse disorder or risk factors, medical comorbidities, and potential adverse drug interactions must be closely monitored. A multidisciplinary approach is beneficial and provides further insight into the obstacles of opioid use and misuse. This team includes, but is not limited to, medical and interventional pain specialists, physical and occupational therapy, psychiatrists and psychologists, and palliative care specialists. Safe opioid prescribing and monitoring techniques need to be implemented.

1. Current Opioid Misuse Measure is a nine-question patient assessment tool designed to identify drug misuse during chronic opioid therapy; it is commonly used for long-term opioid treatment [33-35].

2. Opioid Risk Tool is a five-question screening tool to predict the risk of drug misuse; it is widely used and included in smartphone apps, increasing its accessibility [36-38].

3. Screener and Opioid Assessment for Patients with Pain-Revised is a 24-question tool used to predict drug misuse before initiation of long-term opioid therapy but has lower sensitivity than other tools [33,39-42]. Providers should still consider patient honesty in answering the questions.

4. Urine Drug Monitoring is one of the few measuring tests available to assess opioid misuse. The test includes point of care testing, which is a dipstick; this testing is readily available and inexpensive [43].

Universal precautions to limit non-medical opioid use

Efforts to address the opioid epidemic require proactive partnerships, including healthcare providers, pharmaceutical industries, judicial personnel, emergency management personnel, and government agencies. Addressing the opioid epidemic requires a multidisciplinary approach to increase community knowledge and awareness, educate, and screen to help identify those at risk [28].

Patients and caregivers need to be well informed about treatment with opioids, especially their side effects. Adverse effects may cause opiophobia among patients and caregivers and can trigger misunderstanding of the indications, resulting in inadequate use and/or misuse [44,45]. Without adequate education, patients and caregivers may seek information online or from other non-medical sources. Prescribers also have several misconceptions regarding the law and regulation of opioid use. Some fear prescribing opioid analgesics, which may cause poor quality of life among the cancer patient population [46]. The following serves as an eight-step guideline for the prescription of opioids for cancer patients.

1. Pain diagnosis: assess the pain intensity, location, and causes to determine the need for opioid therapy [47].

2. Screening: using the risk assessment questionnaires to identify the risk of misuse before starting therapy [48].

3. Informed consent: review, in detail, the risks, benefits, and side effects of opioid therapy [49]. The informed consent process should include management of patient and caregiver expectations regarding the management of breakthrough and background pain, with an understanding that the regimen will need to be optimized over time.

4. Treatment agreement: obtain a verbal or written agreement on the therapy plan [50-52]. One should have a pain plan in place with a controlled substance agreement.

5. Review and consider applying adjuvant analgesics to optimize the pain plan better.

6. Implement a multimodal approach, including specialist referral: consider referral of patients at high risk of substance abuse [53-56].

7. Subsequent monitoring: conduct follow-up, urine dipsticks, behavioral assessment, and therapeutic decision-making support [57,58].

8. Pain outcome assessment: assess the effect of the therapy plan.

General principles for prescribing

Pain is the most frequent indication for opioid use in cancer patients; cancer-related pain is reported in 30-50% of patients who receive cancer therapy and 70% of patients suffering from advanced cancer.

Pain in cancer patients is caused by metastasis, chemotherapy-induced neuropathy or mucositis, radiation-related adverse events such as dermatitis, or local mass effect. Moderate-to-severe pain is considered an indication of opioid use. Providers should assess the pain frequency, intensity duration, and site, as well as evaluate patients to avoid NMOU [49]. Patients being initiated on opioids should be prescribed the lowest starting dose of immediate-release (IR) opioids; if the patient is expected to require long-term treatment, extended-release (ER) opioids should be considered [59].

General principles for titration and maintenance

Mu-agonist opioids are the most used in cancer pain because of the absence of ceiling effects. There is no standard or fixed dose of opioids, with the dosing depending on patients’ needs, tolerability, pain frequency, and age. Younger patients need higher doses than older patients. Titration is necessary during treatment depending on the patient’s response and risk for NMOU.

Parenteral Dose Titration

When using morphine, the previous total dose of morphine given in the last 24 hours is divided by three, and then divided by 24 to provide a continuous hourly infusion. Twice the calculated amount is administered initially, and the hourly dose is repeated every 15 minutes until the pain is controlled.

Oral Dose Titration

The last 24-hour doses are totaled and divided by the preferred frequency in 24 hours [60-62]. Dosing frequency depends on the duration of action of morphine or other opioid formulations given in equivalent doses. Similar to insulin, morphine should be prescribed in a basal-bolus fashion. The calculated 24-hour amount should be changed to an ER formulation, with 10-20% of the dose available for breakthrough pain in an IR formulation [63].

Cancer patients who receive long-term opioid therapy are at risk of developing tolerance, dependence, and NMOU; hence, they need opioid maintenance therapy. Methadone and buprenorphine can be used. Buprenorphine is a semisynthetic opioid, highly bound and partial agonist on Mu receptors, and can be used in renal impairment. Methadone is a synthetic opioid agonist therapy that has an analgesic effect with controlling side effects.

Buprenorphine/naloxone is another combination at a ratio of 4:1 to maintain the therapeutic effect and have some opioid antagonism effect of naloxone, which at a low dose controls the side effects without significant reversal of analgesia [64-67]. Pain management consultation should be requested when utilizing methadone or buprenorphine.

General principles for safety

Opioids have common side effects such as constipation, nausea, sedation, respiratory distress, and opioid-induced neurotoxicity (OIN). OIN consists of sedation, cognitive impairment, delirium, hallucinations, myoclonus, and opioid-induced hyperalgesia. Elderly patients or those using sedatives such as benzodiazepines or antihistamines are at a higher risk of OIN. Considering the side effects and dangers of NMOU and abuse, monitoring must be done frequently using assessment tools, urine drug tests, and routine lab work, as well as reducing daily doses when the pain is controlled [47,68,69].

## Conclusions

Opioid misuse is a common challenge during the management of cancer-related pain during an opioid epidemic. However, it can be monitored and modified. It requires a multidisciplinary approach to pain management, increased awareness and education about opioid therapy, and screening to help identify those at risk for opioid misuse. We propose a multimodal approach involving psychiatry teams and interventional pain management for managing cancer-related pain. Utilizing general pain assessment and prescribing, titration, and maintenance strategies is key to successful pain management and limiting NMOU in the cancer population.

## References

[1] Mattson CL, Tanz LJ, Quinn K, Kariisa M, Patel P, Davis NL (2021). Trends and geographic patterns in drug and synthetic opioid overdose deaths - United States, 2013-2019. MMWR Morb Mortal Wkly Rep.

[2] Chino F, Kamal A, Chino J (2020). Incidence of opioid-associated deaths in cancer survivors in the United States, 2006-2016: a population study of the opioid epidemic. JAMA Oncol.

[3] Eiden C, Ginies P, Nogue E, Damdjy Y, Picot MC, Donnadieu-Rigole H, Peyrière H (2019). High prevalence of misuse of prescribed opioid analgesics in patients with chronic non-cancer pain. J Psychoactive Drugs.

[4] Jairam V, Yang DX, Verma V, Yu JB, Park HS (2020). National patterns in prescription opioid use and misuse among cancer survivors in the United States. JAMA Netw Open.

[5] Sanford NN, Sher DJ, Butler SS, Xu X, Ahn C, Aizer AA, Mahal BA (2019). Prevalence of chronic pain among cancer survivors in the United States, 2010-2017. Cancer.

[6] Jiang C, Wang H, Wang Q, Luo Y, Sidlow R, Han X (2019). Prevalence of chronic pain and high-impact chronic pain in cancer survivors in the United States. JAMA Oncol.

[7] Bluethmann SM, Mariotto AB, Rowland JH (2016). Anticipating the "silver tsunami": prevalence trajectories and comorbidity burden among older cancer survivors in the United States. Cancer Epidemiol Biomarkers Prev.

[8] Webster LR, Grabois M (2015). Current regulations related to opioid prescribing. PM R.

[9] Vranken MJ, Lisman JA, Mantel-Teeuwisse AK (2016). Barriers to access to opioid medicines: a review of national legislation and regulations of 11 central and eastern European countries. Lancet Oncol.

[10] Berterame S, Erthal J, Thomas J (2016). Use of and barriers to access to opioid analgesics: a worldwide, regional, and national study. Lancet.

[11] Greco MT, Roberto A, Corli O, Deandrea S, Bandieri E, Cavuto S, Apolone G (2014). Quality of cancer pain management: an update of a systematic review of undertreatment of patients with cancer. J Clin Oncol.

[12] Higginson IJ, Gao W (2012). Opioid prescribing for cancer pain during the last 3 months of life: associated factors and 9-year trends in a nationwide United Kingdom cohort study. J Clin Oncol.

[13] Haumann J, Joosten EB, Everdingen MH (2017). Pain prevalence in cancer patients: status quo or opportunities for improvement?. Curr Opin Support Palliat Care.

[14] McDonald J (2013). Opioid prescribing: guidelines, laws, rules, regulations, policies, best practices. R I Med J (2013).

[15] Riley J, Branford R, Droney J (2015). Morphine or oxycodone for cancer-related pain? A randomized, open-label, controlled trial. J Pain Symptom Manage.

[16] Caraceni A, Shkodra M (2019). Cancer pain assessment and classification. Cancers (Basel).

[17] Wong SS, Cheung CW (2020). Optimization of opioid utility in cancer pain populations. Ann Palliat Med.

[18] Bohnert AS, Guy GP Jr, Losby JL (2018). Opioid prescribing in the United States before and after the Centers for Disease Control and Prevention's 2016 opioid guideline. Ann Intern Med.

[19] Jairam V, Yang DX, Pasha S, Soulos PR, Gross CP, Yu JB, Park HS (2021). Temporal trends in opioid prescribing patterns among oncologists in the Medicare population. J Natl Cancer Inst.

[20] Dowell D, Haegerich TM, Chou R (2016). CDC guideline for prescribing opioids for chronic pain--United States, 2016. JAMA.

[21] Westanmo A, Marshall P, Jones E, Burns K, Krebs EE (2015). Opioid dose reduction in a VA health care system--implementation of a primary care population-level initiative. Pain Med.

[22] Seal KH, Rife T, Li Y, Gibson C, Tighe J (2020). Opioid reduction and risk mitigation in VA primary care: outcomes from the integrated pain team initiative. J Gen Intern Med.

[23] Praveen KT, Law F, O'Shea J, Melichar J (2012). Opioid dependence. Am Fam Physician.

[24] Praveen KT, Law F, O'Shea J, Melichar J (2011). Opioid dependence. BMJ Clin Evid.

[25] Perry BL, Pescosolido BA, Krendl AC (2020). The unique nature of public stigma toward non-medical prescription opioid use and dependence: a national study. Addiction.

[26] Strickland JC, Lile JA, Stoops WW (2019). Evaluating non-medical prescription opioid demand using commodity purchase tasks: test-retest reliability and incremental validity. Psychopharmacology (Berl).

[27] Claxton R, Arnold RM (2011). Screening for opioid misuse and abuse #244. J Palliat Med.

[28] Chan K, Moller M, Marsack-Topolewski C, Winston P, Jennings R, Prifti A (2020). Age differences in non-medical prescription opioid use and psychological distress. Subst Use Misuse.

[29] Bicket MC, Park JN, Torrie A, Allen ST, Weir BW, Sherman SG (2020). Factors associated with chronic pain and non-medical opioid use among people who inject drugs. Addict Behav.

[30] Ducharme J, Moore S (2019). Opioid use disorder assessment tools and drug screening. Mo Med.

[31] Portenoy RK, Hagen NA (1990). Breakthrough pain: definition, prevalence and characteristics. Pain.

[32] Li Z, Aninditha T, Griene B (2018). Burden of cancer pain in developing countries: a narrative literature review. Clinicoecon Outcomes Res.

[33] Varney SM, Perez CA, Araña AA (2018). Detecting aberrant opioid behavior in the emergency department: a prospective study using the screener and Opioid Assessment for Patients with Pain-Revised (SOAPP®-R), Current Opioid Misuse Measure (COMM)™, and provider gestalt. Intern Emerg Med.

[34] McCaffrey SA, Black RA, Villapiano AJ, Jamison RN, Butler SF (2019). Development of a brief version of the Current Opioid Misuse Measure (COMM): the COMM-9. Pain Med.

[35] Goedken AM, Butler CM, McDonough RP, Deninger MJ, Doucette WR (2018). Continuous Medication Monitoring (CoMM): a foundational model to support the clinical work of community pharmacists. Res Social Adm Pharm.

[36] Witkin LR, Diskina D, Fernandes S, Farrar JT, Ashburn MA (2013). Usefulness of the opioid risk tool to predict aberrant drug-related behavior in patients receiving opioids for the treatment of chronic pain. J Opioid Manag.

[37] Webster LR, Webster RM (2005). Predicting aberrant behaviors in opioid-treated patients: preliminary validation of the Opioid Risk Tool. Pain Med.

[38] Jones T, Passik SD (2011). A comparison of methods of administering the opioid risk tool. J Opioid Manag.

[39] Yasin JT, Leader AE, Petok A, Garber G, Stephens B, Worster B (2019). Validity of the screener and opioid assessment for patients with pain-revised (SOAPP-R) in patients with cancer. J Opioid Manag.

[40] Finkelman MD, Smits N, Kulich RJ (2017). Development of short-form versions of the Screener and Opioid Assessment for Patients with Pain-Revised (SOAPP-R): a proof-of-principle study. Pain Med.

[41] Finkelman MD, Kulich RJ, Butler SF, Jackson WC, Friedman FD, Smits N, Weiner SG (2016). An investigation of completion times on the Screener and Opioid Assessment for Patients with Pain - revised (SOAPP-R). J Pain Res.

[42] Black RA, McCaffrey SA, Villapiano AJ, Jamison RN, Butler SF (2018). Development and validation of an eight-item brief form of the SOAPP-R (SOAPP-8). Pain Med.

[43] Arthur JA (2020). Urine drug testing in cancer pain management. Oncologist.

[44] Morgan JP (1985). American opiophobia: customary underutilization of opioid analgesics. Adv Alcohol Subst Abuse.

[45] Zylicz Z (1993). Opiophobia and cancer pain. Lancet.

[46] Charalambous A, Zorpas M, Cloconi C, Kading Y (2019). Healthcare professionals' perceptions on the use of opioid analgesics for the treatment of cancer-related pain in Cyprus: a mixed-method study. SAGE Open Med.

[47] Dalal S, Bruera E (2012). Assessing cancer pain. Curr Pain Headache Rep.

[48] Hui D, Abdelghani E, Chen J (2020). Chronic non-malignant pain in patients with cancer seen at a timely outpatient palliative care clinic. Cancers (Basel).

[49] Dalal S, Tanco KC, Bruera E (2013). State of art of managing pain in patients with cancer. Cancer J.

[50] Savage S (2010). The patient-centered opioid treatment agreement. Am J Bioeth.

[51] Ghods MP, Schmid IT, Pamer CA, Lappin BM, Slavin DC (2015). Developing and initiating validation of a model opioid patient-prescriber agreement as a tool for patient-centered pain treatment. Patient.

[52] Fishman SM, Gallagher RM, McCarberg BH (2010). The opioid treatment agreement: a real-world perspective. Am J Bioeth.

[53] Soares LG, Chan VW (2007). The rationale for a multimodal approach in the management of breakthrough cancer pain: a review. Am J Hosp Palliat Care.

[54] Patarica-Huber E, Boskov N, Pjevic M (2011). Multimodal approach to therapy-related neuropathic pain in breast cancer. J BUON.

[55] Lee CS, Park SJ, Hong SH (2021). Clinical effect of multimodal perioperative pain management protocol for minimally invasive colorectal cancer surgery: propensity score matching study. Asian J Surg.

[56] Hinther A, Nakoneshny SC, Chandarana SP (2021). Efficacy of multimodal analgesia for postoperative pain management in head and neck cancer patients. Cancers (Basel).

[57] Sohn M, Talbert JC, Huang Z, Oser C, Freeman PR (2021). Trends in urine drug monitoring among persons receiving long-term opioids and persons with opioid use disorder in the United States. Pain Physician.

[58] Henry SG, Stewart SL, Murphy E (2021). Using prescription drug monitoring program data to assess likelihood of incident long-term opioid use: a statewide cohort study [In Press]. J Gen Intern Med.

[59] Dalal S, Hui D, Nguyen L, Chacko R, Scott C, Roberts L, Bruera E (2012). Achievement of personalized pain goal in cancer patients referred to a supportive care clinic at a comprehensive cancer center. Cancer.

[60] Walsh TD (1990). Continuing care in a medical center: the Cleveland Clinic Foundation Palliative Care Service. J Pain Symptom Manage.

[61] Walsh D, Rivera NI, Davis MP, Lagman R, Legrand SB (2004). Strategies for pain management: Cleveland Clinic Foundation guidelines for opioid dosing for cancer pain. Support Cancer Ther.

[62] Goldstein P, Walsh D, Horvitz LU (1996). The Cleveland Clinic Foundation Harry R. Horvitz Palliative Care Center. Support Care Cancer.

[63] Hadler R, Moryl N (2019). Pain medications. Pocket Oncology.

[64] Stimmel B (2001). Maintenance therapy for opioid addiction with methadone, LAAM and buprenorphine: the Emperor's New Clothes Phenomenon. J Addict Dis.

[65] Soyka M, Apelt SM, Lieb M, Wittchen HU (2006). One-year mortality rates of patients receiving methadone and buprenorphine maintenance therapy: a nationally representative cohort study in 2694 patients. J Clin Psychopharmacol.

[66] Fontaa V, Bronner C (2001). [Persistent injecting practices in subjects on methadone or buprenorphine maintenance therapy. A study of 600 cases]. Ann Med Interne (Paris).

[67] Alford DP, Compton P, Samet JH (2006). Acute pain management for patients receiving maintenance methadone or buprenorphine therapy. Ann Intern Med.

[68] Dalal S, Bruera E (2009). Cancer pain - progress and ongoing issues in the United States. Pain Res Manag.

[69] Dalal S, Bruera E (2013). Access to opioid analgesics and pain relief for patients with cancer. Nat Rev Clin Oncol.

